# Comment on the 'Oncoplastic Breast Reconstruction with Omental Flap: A Retrospective Study and Systematic Review'. The Omental Flap can be an excellent choice for oncoplastic Breast Reconstruction

**DOI:** 10.7150/jca.35649

**Published:** 2020-06-21

**Authors:** Tert C. van Alphen, Maarten R. Fechner, Coralien L. Broekhuysen

**Affiliations:** Máxima Medical Center, Department of Plastic Surgery, De Run 4600, 5504DB. Veldhoven, the Netherlands

We have read with great interest the retrospective study and systematic review of oncoplastic breast reconstruction with omental flap by Ni et al.^1^ The authors present a case series of 7 patients that underwent a laparoscopically harvested omental free flap (LHOFF) for breast reconstruction. The authors also performed a systematic review where they discuss 15 articles reporting the use of the omentum for breast reconstruction. In these 15 articles, only five articles reported the use of a free omental flap which was used in 37 of the 410 reported cases. We want to congratulate the authors for this successful review article and make some contributions.

In the review article, the authors also included our case series of 6 LHOFF 'The laparoscopically harvested omentum as a free flap for autologous breast reconstruction' published in Microsurgery by van Alphen et al.^2^ We notice that Ni et al. extracted different data or misinterpret our study which we would like to discuss.

At first, Ni et al. have awarded us too much honor. They cited that we 'van Alphen et al.' first described the technique in the 1880s but it was Senn, described the pedicled omental flap for the first time while using it to protect a sutures line of an intestinal anastomosis.^3^ In table 2 of the review, the researchers state that our flap design was a pedicle design, but we only use the omentum in a free flap design, the LHOFF. In table 4 the authors wrongly state about the satisfactory cosmetic results; all patients were satisfied with the results (4 very satisfied and 2 satisfied).

The review also reported that we had flap necrosis as a complication. We did have a necrosis as a complication, but it was a different type of necrosis: skin flap necrosis. Flap necrosis did not occur in our case series. And finally, it is mentioned that our group did not report the time of the hospital stay, but in our paper, we state that patients were hospitalized four to five days.

We are pleased that Ni et al. performed this systematic review of oncoplastic breast reconstruction with the omental flap. Our opinion is that the LHOFF can be an excellent option for autologous reconstructions and that this technique deserves more attention in the current options of autologous breast reconstruction. The aesthetic results are pleasing with minimal scarring, good volume, and a soft, natural feeling breast.
